# The Effect of Ethanolic Extract of Lippia Citriodora on Rats with Chronic Constriction Injury of Neuropathic pain

**DOI:** 10.22074/cellj.2018.4481

**Published:** 2017-11-04

**Authors:** Bahareh Amin, Reyhaneh Noorani, Bibi Marjan Razavi, Hossein Hosseinzadeh

**Affiliations:** 1 Cellular and Molecular Research Center, Department of Physiology and Pharmacology, Faculty of Medicine, Sabzevar University of Medical Sciences, Sabzevar, Iran; 2 Department of Pharmacodynamics and Toxicology, Pharmaceutical Research Center, School of Pharmacy, Mashhad University of Medical Sciences, Mashhad, Iran; 3 Targeted Drug Delivery Research Center, Department of Pharmacodynamy and Toxicology, School of Pharmacy, Mashhad University of Medical Sciences, Mashhad, Iran

**Keywords:** Apoptosis, Astroglia, *Lippia*, Microglia, Neuropathic Pain

## Abstract

**Objective:**

We examined the protective effects of ethanolic extract of *Lippia citriodora (L. citriodora)* on rats subjected to chronic constriction injury (CCI) of sciatic nerve and possible mechanisms of actions.

**Materials and Methods:**

In this experimental study, the extract was administered 50, 100 and 200 mg/kg, Intraperitoneally (I.P) from the surgery time for 14 consecutive days. The changes in the spinal cord levels of apoptotic factors, microglia and astroglia markers during the time course of study were assessed by western blotting on days 3, 7 and 14 post-CCI.

**Results:**

CCI rats developed neuropathy evident from a marked mechanical allodynia, cold allodynia and thermal hyperalgesia on days 3, 5, 7, 10 and 14 post-CCI. A significant increase in the levels of Iba (a marker of microglia activation) and Bax (a proapoptotic factor) was observed three days after nerve injury. The levels of Iba remained high on day 7. In contrast, there was no difference in glial fibrillary acidic protein (GFAP) contents between sham and CCI animals. Treatment with the extract significantly attenuated behavioral changes associated with neuropathy. Bax/Bcl-2 and Iba1 were decreased in CCI animals treated with the extract.

**Conclusion:**

The results support the evidence that microglial activation and apoptosis are correlated with pain behaviors. It is suggested that anti-allodynic and anti-hyperalgesic effects, elicited by *L. citriodora*, might have some degrees of association with the inhibition of microglia activation and apoptotic pathways.

## Introduction

Chronic neuropathic pain often accompanies nerve injury or damage to the nervous system and it can be categorized as central or peripheral pain. Important manifestations are spontaneous pain, allodynia (a painful response to innocuous stimulus), and hyperalgesia (increased sensitivity to nociceptive stimulus). As current therapies have low efficacy and do not adequately alleviate pain (1), further investigations are needed for new pain relieving agents with improved sideeffect profiles. There is nowadays a considerable interest for natural anti-oxidants in the treatment of chronic conditions, such as neuropathic pain (2, 3).

*Lippia* citriodora, Lippia tryphilla or Aloysia tryphilla (verbenaceae), a plant originated from South America, was introduced into Europe at the end of the 17^th^ century and is now adapted well to the Mediterranean area. Based on the region, this plant is variously called, including lemon verbena (English), Lousia (Arabic), and Behlimoo in Persian. The leaves of this plant are used in different types of food and tea due to the lemon-like aroma. Lemon verbena has been widely used in folk medicine for treatment of various ailments, such as fever, cold, skin infections and digestive problems. In addition, lemon verbena has been used based on its anti-spasmodic, anti-pyretic and sedative effects (4, 5). The main components of this popular herb are composed of several iridoids, flavonoids, phenolic acids and phenylpropanoids, particularly verbascoside, as powerful anti-oxidants. *Lippia* extracts and verbascoside, the most abundant constituent, are known to have various pharmacological effects including anti-bacterial (6), antiulcerogenic (7), anti-proliferative (8), anti-oxidant (9-12), as well as learning and memory improvement (13).

Anti-inflammatory and anti-nociceptive effects of this plant have been demonstrated in some studies (14-16). In recent years, more attention has been paid to the role of glial cells (microglia and astroglia) in the pathogenesis of neuropathic pain. Furthermore, a growing body of data indicates that oxidative stress and apoptosis have a role in the induction and development of neuropathic pain (17). In this study, we investigated the effect of 14 days intraperitoneal (I.P) administration of *Lippia citriodora (L. citriodora)* ethanolic extract on the behavioral parameters of rats, undergoing chronic constriction injury (CCI) of neuropathic pain. In addition, the impact of this extract was studied in the protein levels of ionized calcium binding activated protein 1 1 (Iba1), as a marker of microglia activation, glial fibrillary acidic protein (GFAP), as a marker of astroglia activation, as well as two pro-apoptotic and anti-apoptotic proteins, Bcl2-associated X (Bax) and B-cell lymphoma (Bcl2) respectively.

## Materials and Methods

In this experimental study, Ketamine and xylazine (Alfasan Pharmaceutical Co., Switzerland) were I.P injected at dosages of 64 and 1.6 mg/kg, respectively. Gabapentin was a gift from Tehran Darou Pharm Co. (Iran). Ethanolic extract was obtained in our laboratory. It was dissolved in normal saline (0.9%), and administered I.P, immediately after preparation at the dosages of 50, 100 and 200 mg/kg body weight, once a day. Dosage selection was based on the previous studies (2, 5, 18). Administration of the extracts were started immediately after surgery and continued until day 14.

### Extract preparation

Identification of the plant was confirmed by botanists in the Department of Pharmacognosy, Faculty of Pharmacy, Mashhad University of Medical Sciences (Iran). For preparing ethanolic extract, the fine powder dried leaves (200 g) were macerated in successive portions of ethanol (80%, v/v) for 72 hours and the mixture was subsequently filtered and concentrated at 50˚C. The yielded extract was freeze-dried and stored at -20˚C (19).

### High-performance liquid chromatography with a diode array detector analysis

High-performance liquid chromatography-diode array detector (HPLC-DAD) was performed on a KNAUER liquid chromatograph system consisting of a quaternary pump (Smartline Pump 1000, Germany). Detection was carried out using UV-visdiode array detector (Smartline DAD 2800, Germany), and data were processed using Agilent EZChrom Elite software (version 3.3, USA). The ethanolic extract was subjected to reverse-phase HPLC using a gradient method of 20-100% methanol (A) in water as the eluent including 0.05% trifluoroacetic acid. The preparative C18 (5 μl, 21.2×250 mm) and flow rate (10 ml/minute) were used. The linear gradient program was used as follow: 0-10 minute (s), 0-20% methanol; 10- 15 minutes, 20-80% methanol; 15-20 minutes, 80-100% methanol; 20-25 minutes, 100% methanol; 25-28 minutes, 100-20% methanol; 28-30 minutes, 20% methanol. The peaks were monitored at 320 nm (20).

These experiments were carried out on adult male Wistar rats, 250-270 g in weight. The animals were housed on a 12 hours alternating light-dark cycle at a temperature of 22 to 24˚C, in the animal room of the School of Pharmacy, Mashhad University of Medical Sciences. Rats were provided with standard rodent chow and tap water. Experimental protocol was approved by Ethical Committee Mashhad University of Medical Sciences (Approval No.: 910351, 11.Aug. 2012) and conformed to the Internationally Accepted Principles for Laboratory Animal Use and Care (21).

### Induction of neuropathic pain

According to the technique of Bennett and Xie (22), one sciatic nerve of the animals was constricted. Briefly, in rats anesthetized with a cocktail of ketamine and xylazine (64/1.6 mg/kg, I.P), the left sciatic nerve was isolated at the mid-thigh level by blunt dissection, just proximal to its trifurcation and four chromic catgut ligatures (4-0) were applied.

### Study of protocol

87 adult male Wister rats were randomly assigned into following groups: i. Animals without manipulation (naïve animals), ii. CCI animals were treated with normal saline (NS) at a dosage of 1 ml/kg (250 μl for a 250 g animal), iii. Sham group: animals underwent a surgical procedure, without ligature of sciatic nerves, treated with the NS, iv. CCI animals were treated with gabapentin as the reference drug (100 mg/kg) for 14 days, and v-vii. CCI animals were treated with *L. citriodora* ethanolic extract (50, 100 and 200 mg/kg, respectively), administered at dosage of 1 ml/kg for 14 days. On respective days of 3, 7 and 14, the lumbar spinal cord of three animals in each group (except group 3) was rapidly ejected from the vertebral column using a saline-filled syringe, as quickly as possible, and then separated on dry ice. The reason of examining the L4 and L5 segments was based on the consideration that these lumbar segments are the major contributor to the sciatic nerve (22).

### Behavioral tests

In order to establish baseline values of the nociceptive thresholds, pain related behaviors were evaluated at the nerve-damage hind paw, one day before operation as well as at the 3^rd^, 5^th^, 7^th^, 10^th^ and 14^th^ postoperative days. Applied tests were as following order: Von Frey 108 test, acetone and finally thermal stimulus.

### Von Frey hair withdrawal threshold (mechanical allodynia)

A series of calibrated Von Frey filaments (Stoelting, Wood Dale, USA), providing forces of 0.6, 1, 2, 4, 6, 8, 10, 15, 25 and 60 g (cut off=60 grams), were used to measure mechanical sensitivity of the injured hind paw. Each rat was placed in a small plastic cage with a metal mesh floor and allowed to acclimate to their surrounding for 15 minutes before testing. The filaments were repeatedly applied perpendicular to the mid-plantar surface of the hind paw in an ascending stiffness order, until it bent. Each Von Frey filament was applied five times. When rats showed at least three brisk withdrawal responses to a filament, the bending force of the filament was defined as the paw withdrawal threshold (PWT). The hind paw was tested with at least 30 seconds intervals between consecutive stimuli on the injured hind paw (22).

### Acetone drop withdrawal threshold (cold allodynia)

Cold allodynia was assessed using the acetone drop application. Animals were placed in the plexiglas boxes and allowed to acclimatize for about 15 minutes. Acetone test was performed by touching a single bubble of acetone to the mid plantar surface of each injured hind paw from the tip of a 1 ml syringe. A positive response was recorded, if the animal withdraws the paw following application. For each measurement, the paw was sampled five times and a mean PWF was calculated. It was elapsed at least 3 minutes between each test (23).

### Withdrawal latency to a noxious heat stimulus (heat hyperalgesia

A plantar (Hargreaves) analgesic meter (Ugo Basile, Comerio, Italy) (24) was used to assess the latency of response to a noxious heat stimulus applied to the plantar surface of the injured hind paw. Each rat was placed on a 2-mm-thick Plexiglas floor. After 5 minutes of acclimatization to the new environment, the laser radiant heat source was projected to the plantar surface of the hind paw. When animal withdraw its paw, this was detected by a photocell and timer was automatically stopped. The stimulus onset activated a timer which was automatically stopped, upon detecting the evoked paw withdrawal by a photocell. Three latency measurements were taken and averaged for each hind paw during each session of testing. Reduced response of latency to a normally noxious heat stimulus was considered to represent hyperalgesia. Two or three minutes rest was considered between subsequent tests.

### Tissue preparation

After detection of pain threshold on the respective days, three rats from each group were anesthetized and sacrificed by decapitation. For the protein extraction, the lumbosacral spinal cord segment of animals was extracted, stored respectively in liquid nitrogen and -80˚C until usage.

### Western blot assay proteins

At the specified time points, spinal cord tissues were placed in lysis buffer containing 50 mM Tris- HCl (pH=7.4), 2 mM EDTA, 2 mM EGTA, 10 mM NaF, 1 mM sodium orthovanadate (Na_3_VO_4_), 10 mM β-glycerophosphate, 0.2% W/V sodium deoxycholate, 1 mM phenylmethylsulfonyl fluoride (PMSF), and complete protease inhibitor cocktail (Roche, Germany). The homogenate was sonicated on ice with three times of 10-seconds bursts at high intensity with a 10-seconds cooling period between each burst and then centrifuged (10000 g) for 10 minutes at 4˚C. Protein concentration was determined by Bradford assay kit (BioRad, USA) and adjusted (25).

Each adjusted sample was mixed 1:1 v:v with 2x sodium dodecyl sulfate (SDS) blue buffer, boiled, aliquoted and kept at -80˚C. Samples were loaded (50 mg of protein/lane), electrophoresed in a 12% SDS-polyacrylamide gel (SDSPAGE) and blotted to a polyvinylidene fluoride (PVDF) membrane (BioRad, USA). The membranes were incubated overnight at 4˚C with mouse monoclonal anti-GFAP (Cell Signaling, #3670, 1:1000, USA), mouse monoclonal anti- Iba-1 (Santa Cruz #1022-5, 1:1000, USA), rabbit polyclonal anti-Bax (Cell Signaling #2772, 1:1000, USA), rabbit polyclonal anti-Bcl2 (Cell Signaling #2870, 1: 1000, USA) and rabbit polyclonal anti-β-actin antibodies (Cell Signaling #4967, 1: 1000, USA). After washing three times with TBST, membranes were incubated with rabbit horseradish peroxidase-conjugate anti-rabbit IgG (Cell Signaling #7071, 1:2000, USA) or anti-mouse IgG (Cell Signaling #7072, 1:2000, USA). Enhanced chemiluminescence (Pierce, USA) was used to visualize the peroxidase-coated bands. The integral optical density (IOD) of each band was measured using Alliance 4.7 gel-doc (UK), and UVtec software (UK) was used for densitometric analysis of the bands. Protein levels were normalized against intensity of β-actin protein, as control.

### Statistical analysis

All behavioral data are presented as mean ± SEM. Statistical evaluation for behavioral data was made by two-ways ANOVA with repeated measure, followed by Bonferronis’ post hoc test for multiple comparisons. Oneway ANOVA followed by Tukeys’ was used for western blot results. Differences were considered statistically significant, if P<0.05. Statistical analysis was done by Graphpad Prism 6.01 (CA,USA).

## Results

### Chemical analysis

**Fig.1:**
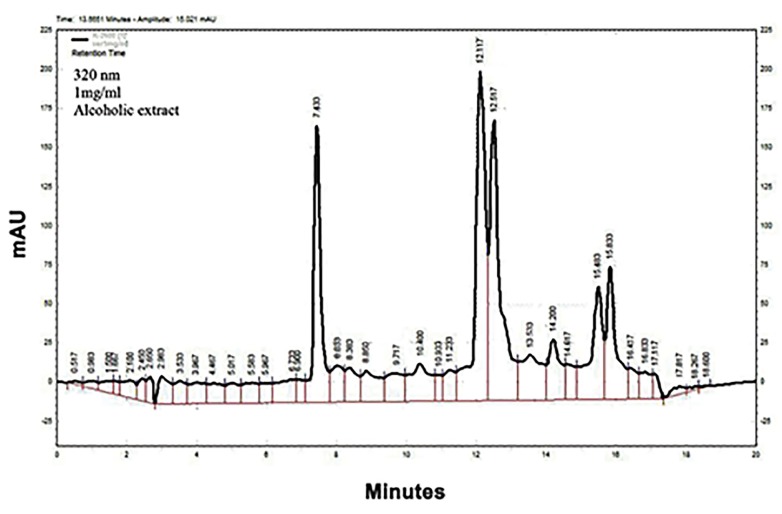
High-performance liquid chromatography (HPLC) fingerprint of the ethanolic extract of L. citriodora on the wavelength of 320 nm.

HPLC fingerprints of the ethanolic extract of *L. citriodora* showed major peaks at the wavelength of 320 nm, for the retention times (minute) of 7.43, 12.11, 12.51, 14.2, 15.48 and 15.83 (Fig .1).

### Effect of *L. citriodora* ethanolic extract on the behavioral signs of neuropathic pain

#### Mechanical allodynia

**Fig.2:**
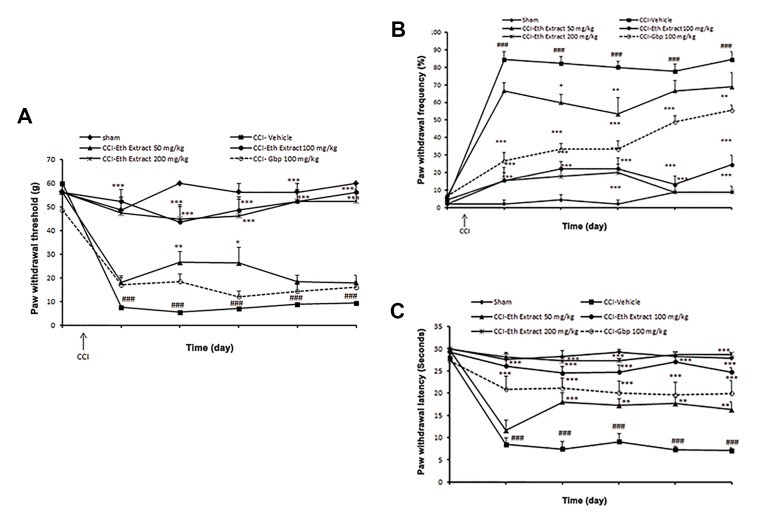
Effect of *L. citriodora* ethanolic extract on the CCI-induced behavioral changes. Extraxt (50, 100 and 200 mg/kg) was administered once per day, from the day of surgery to 14 day. A. Mechano-allodynia in Von-Frey hair test, B. Cold allodynia in acetone drop test, and C. Thermal hyperalgesia in radiant heat test on days -1 (one day before surgery), 3, 5, 7, 10 and 14 after CCI. Rats received either sham or CCI surgery on day 0. Extract was given daily, for 2 weeks from the day of surgery. Values are mean ± SEM, n=9 rats per group; data were analyzed by two-way ANOVA, followed by Bonferronis’ post hoc test ###; P<0.001 NS-CCI animals vs. sham group, *; P<0.05, **; P<0.01, ***; P<0.001 extract vs. NS-CCI animals (control group). Gabepentin (Gbp) is the reference drug, NS; Normal saline, and CCI; Chronic constriction injury.

Assessment of behavioral signs of sham-operated animals indicated that they were statistically indistinguishable from naive animals (data not shown). All of the rats had undergone sciatic nerve CCI, exhibited decreased thresholds of mechanical allodynia in the affected hind paw, 3 days after surgery, as compared to sham animals (7.5 ± 0.64 g vs. 48.7 ± 5.62 g, P<0.001 by two-ways ANOVA). Low dose of ethanolic extract (50 mg/kg) did not modify CCI-induced mechanical hypersensitivity to Von Frey filaments. Mechanical allodynia-like behavior was significantly attenuated in 100 and 200 mg/kg of ethanolic extract, compared to control group on 3-14 days post-operation (P<0.001). However, gabapentin at the administrated dose (100 mg/kg) was not able to efficiently alleviate mechanical allodynia after nerve injury (Fig .2A).

#### Cold allodynia

By 3 days post-CCI, cold allodynia was developed in the ipsilateral paw CCI-NS animals by elevating paw withdrawal frequencies, as compared to sham animals (84.4 ± 4.4% vs. 4.8 ± 2.9%, P<0.001 by two-ways ANOVA). Low dosage of ethanolic extract (50 mg/ kg) did not modify CCI-induced cold allodynia. Cold hypersensitivity was reduced in treated animals with 100 and 200 mg/kg ethanolic extract, on days 3-14 postoperation, as that observed with gabapentin (100 mg/kg) as the reference drug (Fig .2B).

#### Heat hyperalgesia

Three days after CCI, ipsilateral paws exhibited pronounced thermal hyperalgesia for 14 days after the injury, compared to the sham animals (8.5 ± 1.48 seconds vs. 27.6 ± 1.47 seconds, P<0.001 by two-ways ANOVA) (Fig .2C). As did reference drug gabapentin, ethanolic extract (50, 100 and 200 mg/kg) attenuated mean withdrawal latency to thermal stimulus, on days 3-14 after nerve injury.

### Effect of *L. citriodora* ethanolic extract on the spinal cord Bax and Bcl-2 proteins

**Fig.3:**
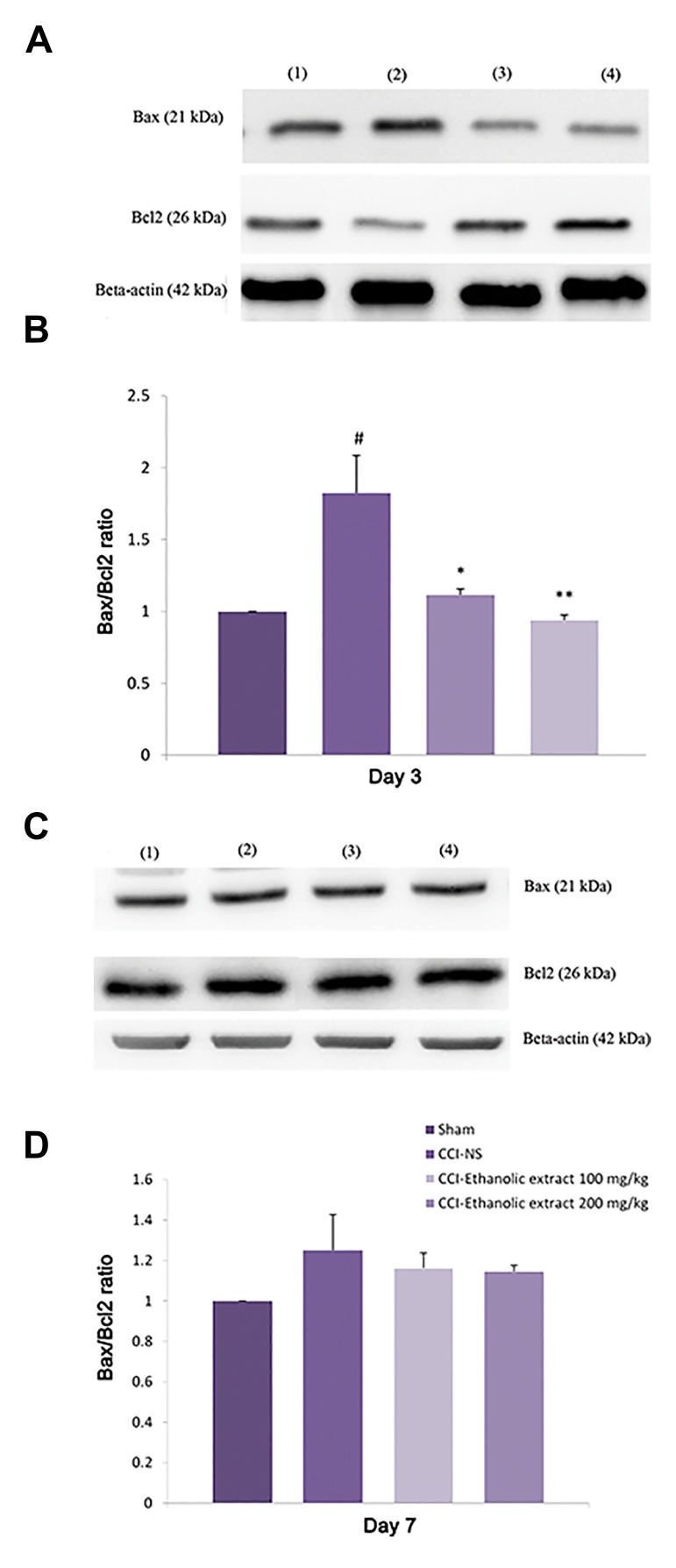
Effect of L. citriodora ethanolic extract on the expression of Bax and Bcl-2 proteins in lumbar spinal cord of chronic constriction injury (CCI) animals. Extraxt (50, 100 and 200 mg/kg) was administered once per day, from the day of surgery to 14 day. A, C. Representative western blot of Bax/Bcl2 on days 3 and 7 respectively, B, and D. Analysis was carried out as described under methods and the bars depict densitometry analyses of western blots from three independent experiments. β-actin (42 kDa) is the loading control protein. Values are mean ± SEM. Data were analyzed by one-way ANOVA, followed by Tukeyss’ post hoc test. #; P<0.01, normal saline (NS)-CCI animals vs. sham group, **; P<0.01, and *; P<0.05, extracts vs. NS-CCI animals (control group).

The expression of spinal cord Bax protein was increased in NS-CCI animals, 3 days after injury (P<0.01), while no obvious change was found in the level of Bcl-2 protein as compared to the sham animals. Hence a significant increase in the Bax: Bcl-2 protein was detected at this time (Fig.3A, B). Treatment of rats with 100 and 200 mg/kg *L. citriodora* ethanolic extract significantly decreased Bax and increased Bcl-2 protein levels on day 3. As a result, the ratio of Bax: Bcl-2 was attenuated in *L. citriodora*-CCI-animals (P<0.01 and P<0.05 for 100 and 200 mg/kg, respectively). Bcl-2 protein content was increased on day 7 in NS-CCI animals (Fig .3C, D). Although Bcl-2 remained elevated in CCI animals receiving *L. citriodora* ethanolic extract, there was no significant difference in the Bax: Bcl-2 ratio among the groups on days 7 and 14 (data not shown) after the injury.

### Effect of L. citriodora ethanolic extract on the spinal cord GFAP protein

**Fig.4:**
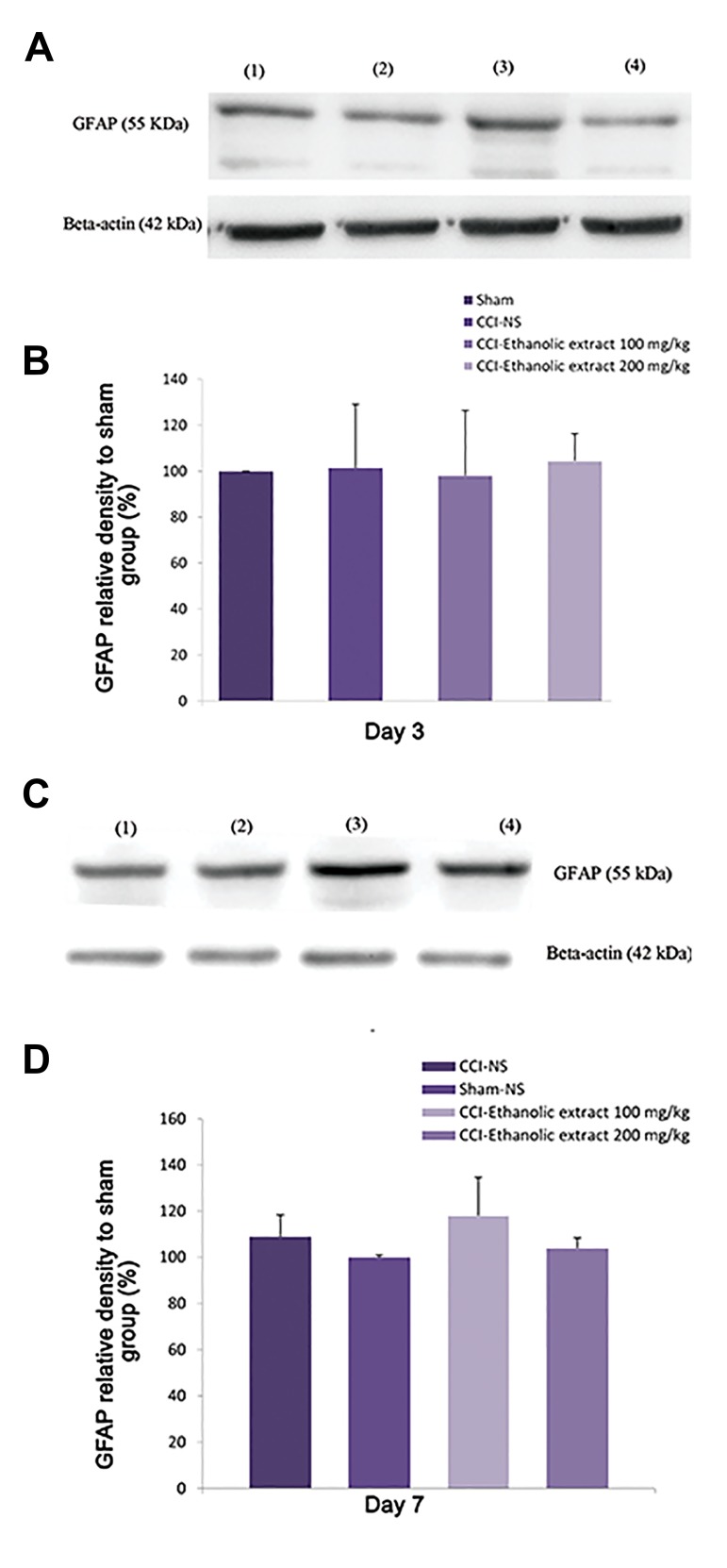
Effect of *L. citriodora* ethanolic extract on the expression of glial fibrillary acidic (GFAP) protein in lumbar spinal cord of CCI animals. Extraxt (50, 100 and 200 mg/kg) was administered once per day, from the day of surgery to 14 day. A, C. Representative western blot of iba1 on days 3 and 7 after CCI, respectively, B, and D. Analysis was carried out as described under methods and the bars depict densitometry analyses of western blots from three independent experiments. β-actin (42 kDa) is the loading control protein. Values are mean ± SEM. Data were analyzed by one-way ANOVA, followed by Tukeyss’ post hoc test.

Western blotting results revealed no significant change in the GFAP levels on days 3 (Fig .4A, B) and 7 (Fig.4C, D) following chronic constriction injury of the sciatic nerve (same results for day 14, data not shown).

### Effect of *L. citriodora* ethanolic extract on the spinal cord Iba protein

**Fig.5:**
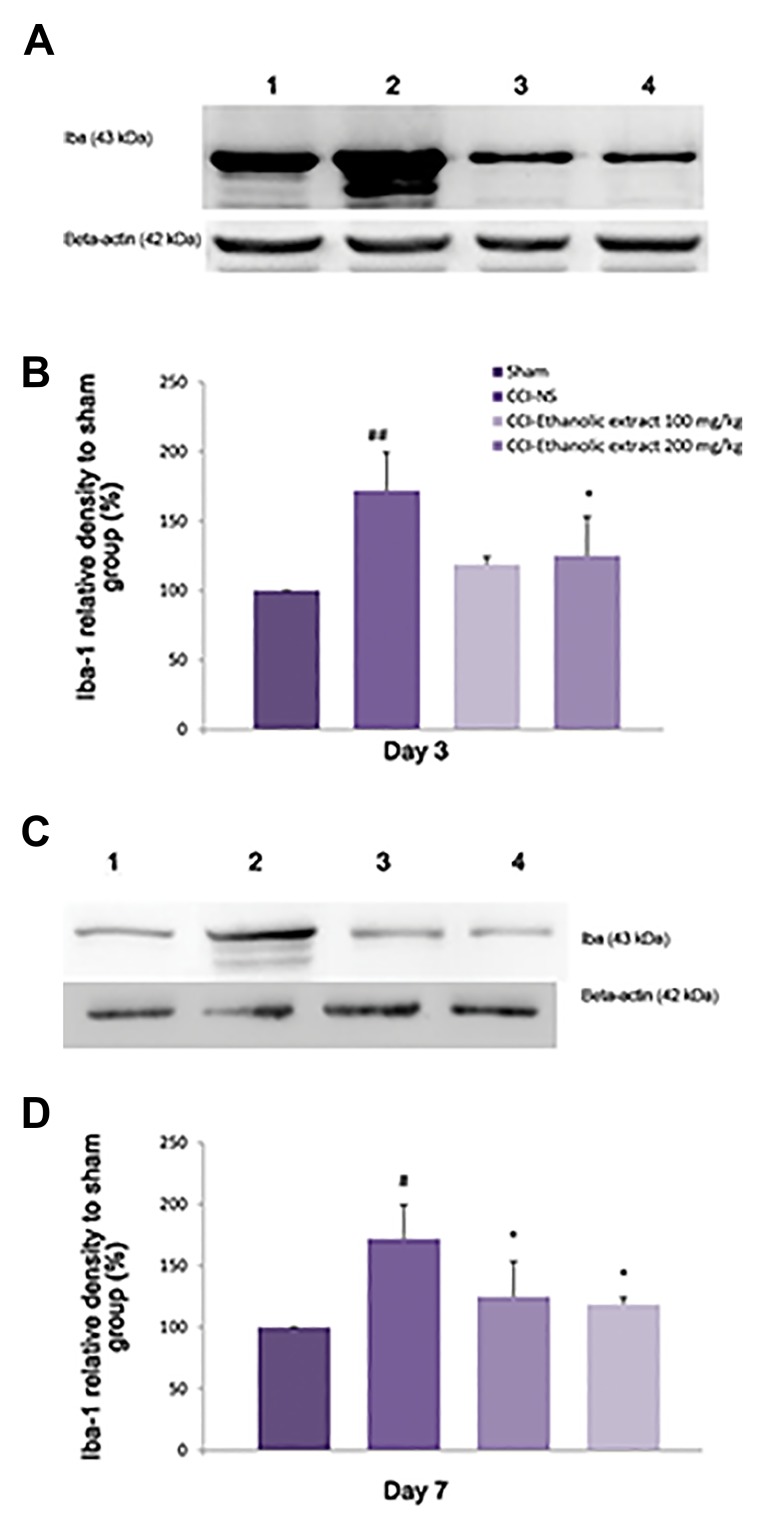
Effect of *L. citriodora* ethanolic extract on the expression of ionized binding (Iba1) protein in lumbar spinal cord of chronic constriction injury (CCI) animals. Extraxt (50, 100 and 200 mg/kg) was administered once per day, from the day of surgery to 14 day. A, C. Representative western blot of iba1 on days 3 and 7 after CCI, B, and D. Analysis was carried out as described under methods and the the bars depict densitometry analyses of western blots from three independent experiments. β-actin (42 kDa) is the loading control protein. Values are mean ± SEM. Data were analyzed by one-way ANOVA, followed by Tukeyss’ post hoc test. ##; P<0.01 NS-CCI animals vs. sham group, #; P<0.05 NS-CCI animals vs. sham group, *; P<0.05 , extracts vs. NS-CCI animals (control group).

On day 3 after CCI, the level of Iba, a marker of microglia activation, was increased in the spinal cord of CCI-NS rats, as compared to sham group (P<0.01, Fig .5A, B). While, this level was declined on day 7 (P<0.05, Fig .5C, D) and day 14 after CCI (data not shown). Treatment with *L. citriodora* ethanolic extract (100 and 200 mg kg) suppressed the activation of microglia 3 and 7 days after surgery, as compared to CCI animals receiving NS (P<0.05 for 100 and 200 mg/kg).

## Discussion

Our data demonstrated that *L. citriodora* ethanolic extract was unable to increase the pain threshold in sham animals, indicating that it has no per se analgesic activity. A period of 14 days intraperitoneal ethanolic extract of *L. citriodora* (100 and 200 mg/ kg) in neuropathic pain rats reduced mechanical allodynia, cold allodynia and thermal hyperalgesia, in comparison with NS-CCI animals which was comparable to reference drug, gabapentin. However, the attenuation of hypersensitivity to innocuous mechanical stimulus was greater than what was observed with gabapentin.

Iba, a marker of spinal microglia activation, was elevated in the spinal cord of animals on days 3 and 7, while it was declined by day 14, following the CCI. The time course of microglial activation was consistent with previous studies (26). Growing body of evidences demonstrated that microglia activation within the spinal cord is key modulator in the central sensitization of neuropathic pain.

Activation of these cells is characterized by morphological modifications and enhanced expression of markers, such as OX-42 and Iba-1 (27, 28). Considering that Iba was increased 3 days after the CCI, it appears that microglia activation parallels the development of neuropathic pain behaviors. In addition, minocycline, a selective inhibitor of microglia activation and pentoxifylline, an inhibitor of cytokine release, prevented mechanical and cold allodynia in the CCI rats, besides the inhibition of spinal cord microglia activation (29). In contrast, there was no difference in the content of GFAP among sham, CCI animals and verbascoside-treated groups 3, 7 or 14 days after surgery. Our data are in agreement with the results of Wodarski et al. (30), indicating that the levels of GFAP did not change after STZ-induced neuropathic pain in diabetic rats. In addition, in an investigation performed by Mika et al. (31), significant up-regulation of OX42 (a microglia marker) and no or little change in GFAP was determined via western blot and immunohistochemical analyses, in the ipsilateral dorsal lumbar spinal cord of CCI rats. A very large increase in the expression of spinal cord microglia marker CD11b/c and no change in the astroglial marker, GFAP, were reported in the morphine-treated CCI mice, as compared to naive animals (32).

However, studies on the activation of GFAP after nerve injury are controversial and require further investigations. There are many studies claiming that astroglia are activated after microglia activation in different kinds of peripheral or central nerve injuries (33, 34). The observed discrepancies might be due to the possibility of astrocyte activation in CCI rat spinal cord at a time point, which was not measured in our study. Le Coz et al. (35) reported while mRNA expression of *Iba* was increased 4 and 21 days after L4-L5 spinal cord injury in three strains of rat, no significant upregulation or difference in GFAP expression was observed in two strains of rat on day 4. Although, a significant GFAP up-regulation was detected 21 days after nerve injury in only Wistar rat.

Another reason for such difference might be due to the variability in the models of induction of neuropathic pain. Most of the studies implicated that similar site for the injury and damage, while in the sciatic nerve CCI, changes are secondary and far from the site of injury. This may explain low activation of spinal astrocytes in the peripheral nerve CCI (36). A marked increase in Bax protein and a slight decrease in Bcl-2 protein level were detected 3 days after CCI. Consequently, Bax/Bcl-2 ratio was significantly increased in animals subjected to the CCI, at this time. Ethanolic extract of *L. citriodora* (100 and 200 mg/kg) for 14 days was capable to normalize the increased Bax/Bcl-2 ratio observed in the CCI animals, at this time. Bax/Bcl-2 ratio was declined thereafter on days 7 and 14, while no difference was detected among NSCCI, sham and neuropathic animals treated with extracts in these days. Our data in the present study supports the evidence that development of neuropathic pain might be associated with the activation of apoptosis process, in the CCI of sciatic nerve (17). The association between neuronal apoptosis in the dorsal horn and the appearance of allodynia or hyperalgesia has not been known yet.

Apoptosis may cause changes in the structure of neurons, resulting in the increased sensitivity of the nociceptive system and subsequently induction of hyperalgesia or allodynia (37). However, there are differences in the pattern of apoptosis occurrence, depending on the time point of study. It seems that as a modulatory mechanism, mitochondrion induced apoptotic process is limited to the first few days after nerve injury. This is in agreement with some of the previous studies. In a study performed by de Novellis et al. (38), an early apoptosis (2-3 days post-CCI) was transiently occurred by the increased ratio of bax/bcl-2 genes. An inversed pattern of bcl-2 family genes expression was detected at later stages. Increased anti-apoptotic expressions, bcl-2 and bcl-xL, resulted in the decreased bax/bcl-2 and bcl-Xs/bcl-xL ratios over the time. As reported by Costa et al. (39), the increased ratio of bax/bcl-2 genes in the spinal cord of CCI rats was also limited to the first few days after nerve injury.

Although the level of pro-apoptotic protein, Bax, was decreased after day 3 and that of microglia marker, Iba, pain related behavior progressively was increased after 7 day, it did not strictly correlate to these factors. Several substances including reactive ROS, cyclooxygenase, nitric oxide (NO) and pro-inflammatory cytokines (TNF-α and IL-1β) are released upon the activation of microglia. All of these factors are implicated in pain facilitation and hence, development and maintenance of chronic pain (40, 41). HPLC-DAD analysis showed 5 major picks at 320 nm, suggesting the presence of phenylpropanoids. This result is accordance with Bilia et al. (20) study, reporting picks at 330 nm for phenylpropanoids (verbascoside and isoverbascoside) and 350 nm for detection of flavonoids. All of these compounds showed anti-oxidant activity in 2,2-diphenyl-1-picrylhydrazyl (DPPH) assay.

Anti-oxidant and anti-inflammatory effects of *L. citriodora* and its bioactive ingredients have been demonstrated in various investigations. In a DPPH assay on the blood of rats, *L. citriodora* increased anti-oxidant enzymes activities, including catalase (CAT), glutathione peroxidase (GPx) and glutathione reductase (GRed). In contrast, it decreased myeloperoxidase (MPO) activity, as a marker of inflammation. The phenylpropanoids verbascoside and isoverbascoside, as well as their metabolites were found to be the main constituents responsible for the anti-oxidant effects of this plant (11). In an *in vitro* model of Parkinsons’ disease, simultaneous treatment with verbascoside, markedly attenuated methyl-4-phenylpyridinium ion induced apoptotic death, oxidative stress and the activation of caspase-3 (42).

Verbascoside was more active than ibuprofen in the acetic acid-induced writhing test and showed similar effects in the tail flick test (16). Studies of Casamassima et al. indicated that plasma oxidative status in lambs supplemented with *L. citriodora* extracts decreased the reactive oxygen metabolites, thiobarbituric acidreactive substances and markedly increased the serum content of vitamins A and E (43). Following treatment of macrophage cells with verbascoside, LPS-induced release of NO and increased levels of the inducible nitric oxide synthase (iNOS) were inhibited (44). Verbascoside has been reported to inhibit apoptosis in galactosamine and lipopolysaccharide-induced liver injury (45). In a recent study on rats subjected to ligature-induced periodontitis, verbascoside reduced cells nuclear factor kappa B (NFkB), a protein complex that controls production of many pro-inflammatory cytokine, iNOS expression, Bax: Bcl-2 ratio, as well as the degree of gingivomucosal tissue injury (46). Although most of beneficial effects of lemon verbena are attributed to verbascoside, other components such as phenolic compounds (i.e. phenolic acids and a flavonoid glycoside, luteolin) were also reported to be responsible for anti-inflammatory effects of *L. citriodora* (47).

Fourteen days after administration of L. citriodora ethanolic extract, tolerance was not determined and the anti-hyperalgesic and anti-allodynic effects were still evident up to the end of study. It is therefore unlikely that relief of the nociceptive responses, induced by verbascoside, could be attributed to opioid receptors. Isacchi et al. (48) also reported that anti-hyperalgesic effect of main bioactive compound, verbascoside, was not prevented by the opioid antagonist naloxone, suggesting that the opioid system has a limited or no role in the antihyperalgesic effects of verbascoside.

## Conclusion

*L. citriodora* could be a good option as an adjunctive therapy in the treatment of neuropathic pain. Antinociceptive effects of ethanolic extract of *L. citriodora* can be attributed, at least in part, to the anti-apoptotic and microglia inhibiting activities.
